
JOURNAL INFORMATION
==============================
NLM Title Abbreviation: City Soc (Wash)
Journal ID: City Soc (Wash)
NLM Journal ID: 101561710
Journal ID: CISO

City & Society (Washington, D.C.)

ISSN: 0893-0465
EISSN: 1548-744X

ARTICLE INFORMATION
==============================
PMCID: PMC7267582
PMID: 32514227
DOI: 10.1111/ciso.12265
Article ID: CISO12265
Article version: 1
Subjects: Original Article, COVID‐19 City Dispatches

Coercion or the social contract? COVID 19 and spatial (in)justice in African cities

Kihato Caroline Wanjiku 1
Landau Loren B 2
1 University of Oxford and University of Witwatersrand
2 University of Oxford and University of Witwatersrand
Electronic publication date: 2020 Apr 28
Print publication date: 2020 Apr
Volume: 32
Issue: 1
Issue title: The Political Materiality of Cities
Electronic Location ID: 10.1111/ciso.12265
Copyright: © 2020 The Authors. City & Society published by Wiley Periodicals LLC on behalf of American Anthropological Association.
License: This is an open access article under the terms of the http://creativecommons.org/licenses/by/4.0/ License, which permits use, distribution and reproduction in any medium, provided the original work is properly cited.
License URL: https://creativecommons.org/licenses/by/4.0/

Figure count: 0
Table count: 0
Page count: 9

source-schema-version-number: 2.0
cover-date: April 2020
details-of-publishers-convertor: Converter:WILEY_ML3GV2_TO_JATSPMC version:5.8.3 mode:remove_FC converted:03.06.2020

[Correction added on May 3, 2020 after first online publication: in the Affiliation section for the author Caroline Wanjiku Kihato, text “Oxford University” has been corrected to “University of Oxford and University of Witwatersrand”.]

==============================
CITY & SOCIETY

*City & Society*

### Coercion or the social contract? COVID 19 and spatial (in)justice in African cities

Caroline Wanjiku Kihato, University of Oxford and University of Witwatersrand

Loren B Landau, University of Oxford and University of Witwatersrand

Still, in every adversity there might be opportunity”

- Jackline Wanyonyi, Slum Dwellers International, Kenya

As the novel corona virus sweeps across the globe, it is giving cause for much reflection. International, national and local policy makers are rethinking the role of aid agencies, and the state, and the relationship between labour and the economy. It is also raising fundamental questions about spatial justice in Africa’s rapidly transforming cities. Advice from the World Health Organisation (WHO) is clear: social distancing and frequent, soapy hand-washing will slow the viral spread. But as commentators have pointed out, adhering to these guidelines is almost impossible when you reside in informal settlements with limited access to water and space (Suttner 2020; Adegbeye 2020; Mitlin 2020). For

© 2020 The Authors. City & Society published by Wiley Periodicals LLC on behalf of American Anthropological Association. This is an open access article under the terms of the Creative Commons Attribution License, which permits use, distribution and reproduction in any medium, provided the original work is properly cited
DOI: 10.1111/ciso.12265

[Correction added on May 3, 2020 after first online publication: in the Affiliation section for the author Caroline Wanjiku Kihato, text “Oxford University” has been corrected to “University of Oxford and University of Witwatersrand".]

millions across sub-Saharan Africa living under just such conditions, WHO's advice is a cruel joke. Accompanied by lock-downs and curfews, the poor must take this advice while struggling harder than ever for the daily wages that put food on the table. Many must decide between viral exposure and starving (Chen 2020; Reddy 2020; Adegbeye 2020). It is Sophie's choice, as a food seller in Abuja articulates: "I cannot afford to stay at home and not feed my children. I know it is risky to be out here, but if I don't come out to look for what to feed my family, we will die of hunger faster than being killed by the virus" (CNN 2020).

And this is just the beginning.

Together with Asia, Africa accounts for 90% of growth in the world's urban population (UNDESA 2018). Estimates are that by between 2010 and 2050, Africa's urban population will triple to 1,23 billion people (UN HABITAT 2010). Places we do not yet consider cities may soon have hundreds of thousands if not a million people. Yet unlike labour intensive industrialisation that drove American and European urbanisation, African cities are characterised by 'jobless urban growth'. Rather than work in factories or offices, the majority of those in cities navigate the precarious informal economy (ILO 2018). Without access to regular income, public housing or credit, majority households are likely to live in settlements that lack tenure security and where they access land, water, sanitation, and housing through informal mechanisms (Tusting et al. 2019). The result is that millions of African urban households are *de facto* part of the city, but live

© 2020 The Authors. City & Society published by Wiley Periodicals LLC on behalf of American Anthropological Association. This is an open access article under the terms of the Creative Commons Attribution License, which permits use, distribution and reproduction in any medium, provided the original work is properly cited
DOI: 10.1111/ciso.12265

[Correction added on May 3, 2020 after first online publication: in the Affiliation section for the author Caroline Wanjiku Kihato, text "Oxford University" has been corrected to "University of Oxford and University of Witwatersrand".]

and work in *de jure* unrecognised and illegal markets and economies. Viewed optimistically, these can be gateways to the cities: spaces of possibility amidst precarity. They are also almost always spaces that can be acted upon or excluded with little political consequence. It is in these sites that that the pandemic reveals the most acute spatial injustices.

Beyond the immediate Faustian bargains between eating and infection, the pandemic exposes and entrenches broader structural inequities. Urban resources do not distribute themselves, their allocations depend on underlying power and decision-making structures that determine who, where and how access is spread across space. More importantly, policy edicts like social distancing, hand washing, lock-downs, curfews and policing (which no doubt save lives) *entrench* these structural injustices. Not only are the urban poor disproportionately affected by urban crises like Covid-19, the policy interventions intended to address them will inadvertently erode what resilience poor households have built to survive. This exacerbates the vulnerability associated with economic and spatial marginalisation.

Already we have evidence of this.

Across Africa's cities, the pandemic is giving governments extraordinary license to evict, lock-down, and control the movements of urban communities. Violent stay at home

© 2020 The Authors. City & Society published by Wiley Periodicals LLC on behalf of American Anthropological Association. This is an open access article under the terms of the Creative Commons Attribution License, which permits use, distribution and reproduction in any medium, provided the original work is properly cited
DOI: 10.1111/ciso.12265

[Correction added on May 3, 2020 after first online publication: in the Affiliation section for the author Caroline Wanjiku Kihato, text "Oxford University" has been corrected to "University of Oxford and University of Witwatersrand".]

orders in Rwanda, South Africa, Kenya, Ghana and elsewhere have seen state security agents shoot dead and publicly flog people caught in the wrong place (Butera 2020; Harding 2020; GhanaWeb 2020; Bearak 2020; Bearak and Ombuor 2020). These actions not only punish transgressors, but also collectively reinforce people's fears of state caprice and coercion. South Africa, ostensibly one of the continent's most progressive states, has already begun plans to 'thin' informal settlement residents in the name of health, (Wicks and Patrick 2020) as activists decry the eviction of informal dwellers in Durban (Abahlali baseMjondolo 2020). If COVID 19 exposes the frailty of urban basic infrastructure and public welfare systems, its mitigation reveals the violent inclinations of its militaries and security forces.

The COVID 19 crisis reveals both the reasons we have governments, and our reasons for fearing them. Without collective action we cannot save lives, but the brutality and repression exercised in the name of saving lives is disquieting. In Accra, simulating prison drills, those caught violating lock-down restrictions are forced to lie prostate and perform calisthenics to punish and humiliate them and broadcast a message to others without the resources to drive or stay at home. In Cape Town's Masiphumelele township, journalist Jacques Marais recounts his encounter with abusive masked the South African Defense Force officers using 4ft long clubs to flog people on the street (Marais 2020). In Kenya, deaths by police have been reported, including a 13 year old boy shot dead by a stray bullet fired by police while enforcing the lock-down. In Durban's working-class

© 2020 The Authors. City & Society published by Wiley Periodicals LLC on behalf of American Anthropological Association. This is an open access article under the terms of the Creative Commons Attribution License, which permits use, distribution and reproduction in any medium, provided the original work is properly cited
DOI: 10.1111/ciso.12265

[Correction added on May 3, 2020 after first online publication: in the Affiliation section for the author Caroline Wanjiku Kihato, text "Oxford University" has been corrected to "University of Oxford and University of Witwatersrand".]

Cato Manor township, South African police arrested twenty-nine women and their children. Their crime was sleeping outside and contravening the lockdown after local authorities ordered their eviction from their homes (Socialist Revolutionary Workers Party 2020).

What the spread and responses to the novel corona virus in poor parts of the city highlight is that geography is not a neutral backdrop in which development activities take place. Rather space is a ‘social product’ (Pirie 1983) that significantly shapes individuals’ and households’ developmental choices and outcomes (Soja 2010). More than this, spatiality and (in)justice are in intrinsically bound with a dialectical mutually reinforcing relationship. Dikeç calls this the ‘spatiality of injustice and the injustice of spatiality’ (Dikeç 2001, 1792). Marginalised communities in African cities face a double disadvantage. For one, they live under conditions prone to spreading disease. They also occupy spaces where compliance with the law is difficult.  Every failure to comply, every violation of an impracticable law or regulation becomes justification for additional restrictions and violence.

Covid-19 is not only a heuristic revealing the precarity of life for millions in cities across the global South.  It also draws attention to the social bases on which people (barely) survive – informal and familial networks, precarious work, seasonal migration, and flexible housing options with limited entry costs. It is these very elements of urban life that now make them particularly vulnerable to disease. But this is nothing new. While

© 2020 The Authors. City & Society published by Wiley Periodicals LLC on behalf of American Anthropological Association. This is an open access article under the terms of the Creative Commons Attribution License, which permits use, distribution and reproduction in any medium, provided the original work is properly cited
DOI: 10.1111/ciso.12265

[Correction added on May 3, 2020 after first online publication: in the Affiliation section for the author Caroline Wanjiku Kihato, text “Oxford University” has been corrected to “University of Oxford and University of Witwatersrand".]

urbanites tend to live longer and healthier lives than similar people elsewhere, conditions are far from ideal. The danger is that these facets of urban life will now be turned against the poor in ways that further their social and geographic marginalisation. In the short-term almost any effort to save lives is potentially justifiable, but the imperatives to protect lives can not come at the cost of long-term livelihoods.

What is needed now are strategies that avoid stigmatising the poor or blaming them for their vulnerability. Regulations and practices exposing the poor as law-breakers and coding them as disease vectors make it more, rather than, less likely that they will be subject to coercion and urban exclusion. Humanitarian politics acts *on* people, ostensibly in their interests. This is no more sustainable than other forms of autocratic rule, especially when such rules are ultimately oriented to preserving existing socio-economic and spatial hierarchies. Instead, interventions justified by Covid-19 should be consultative and forward looking. As the continent's experience with Ebola shows, combatting the pandemic requires strengthening accountability and the social contract between the state and its vulnerable citizens (Christensen et al, 2020).

If this pandemic teaches us anything, it is that the virus has no boundaries, and the wellbeing of both rich and poor are codependent. The only way to disentangle the risk is to remove the poor entirely from cities -- but this is neither practical, possible or just. Rich and poor lives are imbricated within Africa's urban economies. To quote Wanyonyi (2020), 'this adversity presents us an opportunity' to erase the acute inequalities in the

© 2020 The Authors. City & Society published by Wiley Periodicals LLC on behalf of American Anthropological Association. This is an open access article under the terms of the Creative Commons Attribution License, which permits use, distribution and reproduction in any medium, provided the original work is properly cited
DOI: 10.1111/ciso.12265

[Correction added on May 3, 2020 after first online publication: in the Affiliation section for the author Caroline Wanjiku Kihato, text "Oxford University" has been corrected to "University of Oxford and University of Witwatersrand".]

distribution of urban resources in cities. With hope it may encourage policy makers to act with the urban poor rather than 'for' or 'on' them. What is clear is that we can not create the inclusive cities called for by the SDGs and the New Urban Agenda without a more just spatial politics.

References

Abahlali baseMjondolo. 2020. "Abahlali BaseMjondolo | Umhlaba Izindlu NeSithunzi Land Housing Dignity." April 3, 2020. http://abahlali.org/.

Adegbeye, OluTimehin. 2020. "Why Social Distancing Won't Work for Us." The Correspondent. March 27, 2020. https://thecorrespondent.com/378/why-social-distancing-wont-work-for-us/50039243100-5409cfb5.

Bearak, Max. 2020. "Kenya's Coronavirus Curfew Begins with Wave of Police Crackdowns - The Washington Post." *Washington Post*, March 28, 2020, Online edition. https://www.washingtonpost.com/world/africa/kenyas-coronavirus-curfew-begins-with-wave-of-police-crackdowns/2020/03/28/358327aa-7064-11ea-a156-0048b62cdb51_story.html.

Bearak, Max, and Rael Ombuor. 2020. "Kenyan Police Shot Dead a Teenager on His Balcony during a Coronavirus Curfew Crackdown." Washington Post. March 31, 2020. https://www.washingtonpost.com/world/africa/kenyan-police-shot-dead-a-teenager-on-his-balcony-during-a-coronavirus-curfew-crackdown/2020/03/31/6344c70e-7350-11ea-ad9b-254ec99993bc_story.html.

© 2020 The Authors. City & Society published by Wiley Periodicals LLC on behalf of American Anthropological Association. This is an open access article under the terms of the Creative Commons Attribution License, which permits use, distribution and reproduction in any medium, provided the original work is properly cited
DOI: 10.1111/ciso.12265

[Correction added on May 3, 2020 after first online publication: in the Affiliation section for the author Caroline Wanjiku Kihato, text "Oxford University" has been corrected to "University of Oxford and University of Witwatersrand".]

Butera, Saul. 2020. “Rwanda Police Shoot 2; Say Officers Attacked on Patrol.” *Bloomberg*, March 25, 2020, Online edition. https://www.bloomberg.com/news/articles/2020-03-25/rwanda-police-shoot-two-say-officers-attacked-on-patrol.

Chen, Marty. 2020. “To Die from Hunger or the Virus: An All Too Real Dilemma for the Poor in India (and Elsewhere).” Women in Informal Employment: Globalizing and Organizing. *WIEGO Blogs* (blog). April 2020. https://www.wiego.org/blog/die-hunger-or-virus-all-too-real-dilemma-poor-india-and-elsewhere?utm_source=WIEGO&utm_campaign=776d31f892-EMAIL_CAMPAIGN_2020_03_30_03_33+ENG&utm_medium=email&utm_term=0_8ef746a546-776d31f892-330771373.

Christensen, Darin, Oeindrila Dube, Johannes Haushofer, Bilal Siddiqi, and Maarten Voors. 2020. *Building Resilient Health Systems: Experimental Evidence from Sierra Leone and the 2014 Ebola Outbreak*. Los Angeles: Unpublished Paper. https://www.ssrn.com/abstract=3562391.

CNN. 2020. “‘We Don’t Work, We Don’t Eat’: Informal Workers Face Stark Choices as Africa’s Largest Megacity Shuts Down.” *CNN* (blog). March 31, 2020. https://currently.att.yahoo.com/att/dont-dont-eat-informal-workers-131530401.html.

Dikeç, Mustafa. 2001. “Justice and the Spatial Imagination.” *Environment and Planning A* 33 (10): 1785–1805.

© 2020 The Authors. City & Society published by Wiley Periodicals LLC on behalf of American Anthropological Association. This is an open access article under the terms of the Creative Commons Attribution License, which permits use, distribution and reproduction in any medium, provided the original work is properly cited
DOI: 10.1111/ciso.12265

[Correction added on May 3, 2020 after first online publication: in the Affiliation section for the author Caroline Wanjiku Kihato, text “Oxford University” has been corrected to “University of Oxford and University of Witwatersrand".]

GhanaWeb. 2020. "Lockdown: Ghana Police Urges Public to Ignore Viral Brutality Videos." April 1, 2020. https://www.ghanaweb.com/GhanaHomePage/NewsArchive/Lockdown-Ghana-Police-urges-public-to-ignore-viral-brutality-videos-910924.

Harding, Andrew. 2020. "South Africa's Ruthlessly Efficient Fight against Coronavirus." *BBC News*, April 3, 2020, sec. Africa. https://www.bbc.com/news/world-africa-52125713.

ILO. 2018. *Women and Men in the Informal Economy: A Statistical Picture. Third Edition*. Geneva. http://www.ilo.org/global/publications/books/WCMS_626831/lang--en/index.htm.

Marais, Jacques. 2020. "Covid-19 Personal Experience: SANDF and Police's Violent Easter Gift to Masiphumelele." Daily Maverick. April 10, 2020. https://www.dailymaverick.co.za/article/2020-04-10-sandf-and-polices-violent-easter-gift-to-masiphumelele/.

Mitlin, Diana. 2020. "Dealing with COVID-19 in the Towns and Cities of the Global South." IIED. *International Institute for Environment and Development* (blog). March 27, 2020. https://www.iied.org/dealing-covid-19-towns-cities-global-south.

Pirie, Gordon H. 1983. "On Spatial Justice." *Environment and Planning A* 15 (4): 465–473.

© 2020 The Authors. City & Society published by Wiley Periodicals LLC on behalf of American Anthropological Association. This is an open access article under the terms of the Creative Commons Attribution License, which permits use, distribution and reproduction in any medium, provided the original work is properly cited
DOI: 10.1111/ciso.12265

[Correction added on May 3, 2020 after first online publication: in the Affiliation section for the author Caroline Wanjiku Kihato, text "Oxford University" has been corrected to "University of Oxford and University of Witwatersrand".]

Reddy, Sanjay G. 2020. “The Coronavirus Pandemic and the End of Economics?” *Foreign Policy*, March 31, 2020, Online edition, sec. Argument. https://foreignpolicy.com/2020/03/31/coronavirus-pandemic-rethinking-economics/.

Socialist Revolutionary Workers Party. 2020. “Solidarity with Abahlali Basemjondolo.” April 10, 2020. https://www.facebook.com/OfficialSRWP/?__tn__=kC-R&eid=ARCWLXkUmVZ0M_bEQ4sWFPC6Z-.

Soja, Edward W. 2010. “The City and Spatial Justice.” In *Justice et Injustices Spatiales*, edited by Bernard Bret, Philippe Gervais-Lambony, Claire Hancock, and Frédéric Landy, 56–72. Presses universitaires de Paris Ouest. https://doi.org/10.4000/books.pupo.415.

Suttner, Raymond. 2020. “Covid-19: Inequality and Violence.” *Daily Maverick*, April 8, 2020, Online edition, sec. OP-ED. https://www.dailymaverick.co.za/article/2020-04-08-covid-19-inequality-and-violence/.

Tusting, Lucy S., Donal Bisanzio, Graham Alabaster, Ewan Cameron, Richard Cibulskis, Michael Davies, Seth Flaxman, et al. 2019. “Mapping Changes in Housing in Sub-Saharan Africa from 2000 to 2015.” *Nature* 568 (7752): 391–94. https://doi.org/10.1038/s41586-019-1050-5.

UN HABITAT. 2010. *The State of African Cities 2010: Governance, Inquality and Urban Land Markets*. Nairobi.

UNDESA. 2018. *World Urbanization Prospects: The 2018 Revision*. New York: United Nations Department of Economic and Social Affairs. https://population.un.org/wup/Publications/Files/WUP2018-KeyFacts.pdf.

© 2020 The Authors. City & Society published by Wiley Periodicals LLC on behalf of American Anthropological Association. This is an open access article under the terms of the Creative Commons Attribution License, which permits use, distribution and reproduction in any medium, provided the original work is properly cited
DOI: 10.1111/ciso.12265

[Correction added on May 3, 2020 after first online publication: in the Affiliation section for the author Caroline Wanjiku Kihato, text “Oxford University” has been corrected to “University of Oxford and University of Witwatersrand".]

Wanyonyi, Jackline. 2020. "Coming Home to the New Normal: A Covid-19 Story." Muungano wa Wanavijiji. *Muungano Wa Wanavijiji* (blog). April 1, 2020. https://www.muungano.net/browseblogs/2020/4/1/coming-home-to-the-new-normal-a-covid-19-story.

Wicks, Jeff, and Alex Patrick. 2020. "Provinces Brace for 'thinning' of Settlements in Covid-19 Fight." *Sunday Times*, April 4, 2020, Online edition, sec. News. https://www.timeslive.co.za/sunday-times/news/2020-04-05-provinces-brace-for-thinning-of-settlements-in-covid-19-fight/.

© 2020 The Authors. City & Society published by Wiley Periodicals LLC on behalf of American Anthropological Association. This is an open access article under the terms of the Creative Commons Attribution License, which permits use, distribution and reproduction in any medium, provided the original work is properly cited
DOI: 10.1111/ciso.12265

[Correction added on May 3, 2020 after first online publication: in the Affiliation section for the author Caroline Wanjiku Kihato, text "Oxford University" has been corrected to "University of Oxford and University of Witwatersrand".]
